# Osteomyelitis in Cat-Scratch Disease: A Never-Ending Dilemma—A Case Report and Literature Review

**DOI:** 10.1155/2018/1679306

**Published:** 2018-04-01

**Authors:** D. Donà, L. Nai Fovino, E. Mozzo, G. Cabrelle, G. Bordin, R. Lundin, C. Giaquinto, T. Zangardi, O. Rampon

**Affiliations:** ^1^Division of Paediatric Infectious Diseases, Department for Woman and Child Health, University of Padua, Padua, Italy; ^2^Pediatric Emergency Department, Department for Woman and Child Health, University of Padua, Padua, Italy; ^3^PENTA Foundation, Padua, Italy

## Abstract

**Background:**

We performed a review of published case studies of osteomyelitis associated with cat-scratch disease to consolidate existing information on clinical presentation, diagnostic tools, therapy, and outcome, as well as presenting a case of disseminated cat-scratch disease in a 12-year-old female with skull osteomyelitis and spleen involvement.

**Methods:**

A search for articles indexed in PubMed, Embase, and Google Scholar was performed with the search terms “*Bartonella*,” “bone,” “osteomyelitis,” “osteolytic,” and “cat-scratch disease” limited to the immunocompetent pediatric population and articles in English.

**Results:**

51 cases were identified. The average age was 7.8 years with equal sex distribution. Fever (84.3%), often with a prolonged course (64.7%), and osteoarticular pain (88.2%) were the most common clinical findings. Lymphadenopathy was present in 64.7% of patients. Vertebral body was mainly involved (51.9%). MRI (50%) and bone scintigraphy (48.1%) were favored to confirm osteomyelitis, while serology was the preferred microbiological diagnostic. Various antibiotics were prescribed in combined or sequential regimens, with median duration of therapy of 23 days. About 12.5% of patients did not receive any treatment. Most patients had excellent prognosis; in particular, all patients not receiving any therapy showed complete recovery and no recurrence of symptoms.

**Conclusions:**

*Bartonella henselae* should be considered in differential diagnosis of localized lymphadentitis. Osteoarticular pain or limitation during cat-scratch disease in children should always be investigated for bone spreading. Owing to good prognosis, invasive procedures to obtain the bone material should be avoided. Serology is the gold standard diagnostic tool and MRI is the best radiographic technique to define bone and surrounding tissue involvement. Treatment represents a never-ending dilemma: surgical intervention or use of antibiotics is still controversial, and more studies are needed to define the best antimicrobial regimen.

## 1. Background

Cat-scratch disease (CSD) is a common zoonosis caused by *Bartonella henselae* [1].

Affected children typically present with lymphadenitis after local cutaneous reaction at the scratch site.

Complications such as Parinaud's oculoglandular syndrome, erythema nodosum, and granulomas in the liver and spleen occur in approximately 10% of immunocompetent children [2]. Bone involvement during CSD is rare, and skull localization is even more unusual [2–5].

We present a case of disseminated CSD with skull osteomyelitis and spleen involvement and perform a review of pediatric case studies of osteomyelitis associated with CSD to consolidate existing information on clinical presentation, diagnostic tools, therapy, and outcome.

## 2. Case Presentation

A 12-year-old girl presented with a 5-day history of fever, right lateral-cervical and submandibular lymphadenopathy, and frontoparietal headache. The past medical history was uneventful, and she had no pets at home and did not travel recently.

Physical examination revealed a 3 cm painful lateral-cervical swelling with overlying erythematous skin. White blood cell count was 16.2 × 10^3^/*μ*L, with 12.8 × 10^3^/*μ*L neutrophils, while C-reactive protein and erythrocyte sedimentation rate were normal. Oropharyngeal swab was negative. Ultrasound (US) confirmed the nature of the swelling (multiple reactive lymph nodes without central colliquation) and showed other reactive lymph nodes in the left cervical and bilateral inguinal regions. The girl was discharged with a 14-day course of amoxicillin-clavulanate, followed by improvement of cervical swelling and reduction of spiking fever.

After two days from the conclusion of antibiotic therapy, the patient presented to the Emergency Department suffering reoccurrence of severe headache and lymphadenopathy and was admitted for further investigation.

Physical examination was unremarkable, except for right cervical swelling and 2 cm left parietal-occipital swelling.

Complete blood cell count and inflammatory markers were normal; IgM and IgG positivity for *Bartonella henselae* was confirmed through immunofluorescence assay. Suspecting CSD, a new medical history was collected revealing that the child used to play with neighbor's kittens.

**Figure 1:**
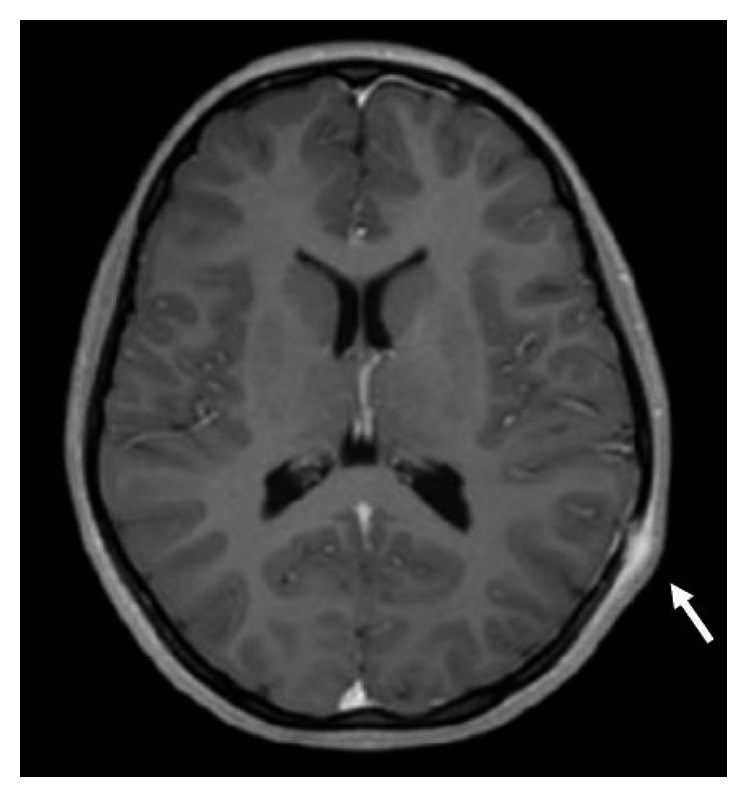
Cranial magnetic resonance imaging with contrast: in the left temporoparietal area in correspondence with clinical painful swelling is present an erosion of the skull of about 10 mm. The external periosteum is thickened with marked contrast enhancement in a 4 cm area.

Due to the clinical finding of scalp swelling, a cranial magnetic resonance imaging (MRI) with contrast was performed, finding an erosion of the skull of about 10 mm in the left temporoparietal area (Figure 1). Other sites of osteomyelitis were excluded by a bone total body scintigraphy. Abdominal showed a round hypoecogenic splenic lesion of 1 cm consistent with a granuloma.

Infectious disease specialists and neurosurgeons advised against surgical intervention, so oral azithromycin was started, with rapid improvement of headache and cranial and cervical swelling. The child was discharged with 4-week therapy of azithromycin.

One month after discharge, cervical swelling and splenic lesion disappeared, but headache persisted with reduced intensity and frequency. Serology was repeated with evidence of IgM negativity and IgG positivity. Eight weeks from symptom onset, MRI was repeated showing persistency of cranial lesion despite antibiotic treatment. Since the girl continued to complain of headache, a 6-week course of oral trimethoprim-sulfamethoxazole (TMP-SMX) and rifampicin was started with gradual clinical improvement. Four months from symptom onset, the osteolytic lesion was undetectable with MRI.

## 3. Methods

Case reports of pediatric osteomyelitis caused by *Bartonella henselae* were reviewed.

A search of English language articles was conducted using PubMed, Embase, and Google Scholar up to 31 January 2016. Search terms were “*Bartonella*,” “bone,” “osteomyelitis,” “osteolytic,” and “cat-scratch disease.”

Only about *Bartonella henselae* bone infections in immunocompetent pediatric populations (≤18 years old) were considered.

To be included, osteomyelitis had to be documented clinically or radiologically during the course of CSD, or *Bartonella henselae* had to be directly detected in the bone lesion.

Demographic data, contact with cats, clinical presentation, laboratory and microbiological tests, radiological findings, treatment (medical and surgical), and outcome were retrieved. Categorical data were summarized as frequency counts and percentages. Continuous data were summarized using the mean and standard deviation or the median and interquartile range, depending on distribution of data.

## 4. Results

The literature review identified 51 eligible pediatric cases of osteomyelitis related to *Bartonella henselae* infection.

All data derived from literature and the current report are summarized in Table 1. A more detailed table can be found as a supplementary material [6–37] (available here).

### 4.1. Demographic and Clinical Characteristics

Mean age at presentation was 7.8 (±3.8) years, with equal sex distribution.

The majority of patients (91.8%) had contact with cats, only one patient (2%) had a dog scratch, and three denied cat contacts (6.1%), while for three children, this information was not available.

Most patients showed fever (84.3%) and 64.7% prolonged course of fever lasting more than 2 weeks. In one case, information about body temperature was not reported.

Lymphadenopathy was present in 64.7% of patients. Sites involved were cervical (45.5%), axillary (15.6%), inguinal (12.1%), epitrochlear (6.1%), submandibular (6.1%), axillary and cervical (3%), preauricular (3%), postauricular (3%), submandibular and axillary (3%), and parasternal (3%). In one case, information about lymphatic involvement was not reported.

Lymphadenopathy was contiguous or in the drainage area of infected bones in only 39.3% of reports.

The most affected bones were vertebral bodies (51.9%) followed by limbs (32.7%), with almost the same distribution between upper and lower limbs (upper limbs 47.1%, lower limbs 52.9%, and both 5.8%). Only 10 cases (19%) (including the current report) presented with skull involvement.

In 73.1% cases, osteomyelitis was unifocal, while in 26.9%, it was multifocal.

At presentation, 88.2% of patients complained of osteoarticular pain, while 37.3% presented with swelling of tissues around the affected bone.

Clinical information including rubor, calor, and functio laesa was not available for two children. Forty percent of patients suffered from functional impairment of the involved osteoarticular area. A minority of children (14%) presented local signs of inflammation including rubor and calor.

Nonspecific signs or symptoms (abdominal pain, fatigue, night sweat, and weight loss) were present in 34.7% of cases (this information was not available for three patients).

Only a few patients had disseminated CSD (16.3%); three of them presented with liver granulomas (one in the context of Parinaud's syndrome), one with isolated spleen lesion, two with the association of hepatic and splenic lesions, one with erythema nodosum, and one with pleural effusion and pneumonia. Only 37.5% of patients with disseminated CSD had multifocal bone involvement.

### 4.2. Diagnosis

Information on inflammatory markers was available for 40 children for ESR and 32 children for CRP. At time of presentation, ESR and CRP were elevated in 85% and 75% of these cases, respectively.

For 51 patients, information existed on microbiological test results.

Skin test was used as a diagnostic tool in 11/40 cases between 1954 and 1994. After 1994, serology was used for CSD diagnosis (78.4% of all cases). Polymerase chain reaction (PCR) was performed in 27.5% of cases: five on bone biopsy, five on aspirated material (abscess/mass), and one on lymph node biopsy. All PCRs were used to confirm a previous positive serology.

All patients showed radiological abnormalities on computed tomography (CT) (46.2%), MRI (50%), bone scintigraphy (48.1%), or X-ray (50%). A combination of radiological exams was used in 65.4% of cases.

Fifteen children received a diagnostic biopsy, eight (53.3%) of bone or mass around bones, five (33.3%) of lymph nodes, one of both lymph node and bone (6.7%), and the remaining of the liver (6.7%).

### 4.3. Treatment, Prognosis, and Outcome

Sixty-six percent of children received only medical therapy, with a wide variety of antibiotic prescriptions reported: macrolides (18/39), rifampicin (13/39), beta-lactams (12/39), TMP-SMX (9/39), aminoglycosides (9/39), quinolones (2/39), clindamycin (2/39), and tetracycline (3/39). In 61.5% of cases, antibiotic therapy was combined or sequential; the most common monotherapies prescribed were macrolides (7/15) followed by beta-lactams (4/15), TMP-SMX (1/15), aminoglycosides (1/15), tetracycline (1/15), and rifampicin (1/15).

The median duration of antibiotic therapy was 23 days (lower to upper quartiles = 20 to 42).

Ten children (20.8%) were treated surgically; for three, the procedure was conclusive, with subsequent antibiotic therapy required for the rest.

12.5% of patients did not receive any treatment, and all showed complete recovery with no recurrence of symptoms.

In four cases, treatment was not described.

No fatal outcomes were reported among the 40 cases with information available on clinical outcome. 43% children experienced rapid resolution of symptoms (<2 weeks from the beginning of symptoms), most of them after antibiotic therapy (64.7%), three (17.6%) with both antibiotic and surgical therapy, and two (11.8%) without therapy.

For patients with prolonged symptoms (57.5%), the median duration was 90 days (lower to upper quartiles = 35 to 180); 65.2% of them received only antibiotic therapy, 26.1% received surgical treatment (in 50% of those cases, it was followed by antibiotic therapy), and 8.7% had no therapy at all.

Information on radiological recovery was available for 69.2% of patients. Most children (63.5%) presented a complete recovery without sequelae within a median of 120 days (lower to upper quartiles = 42 to 240).

Only three patients (7.7%) presented an incomplete recovery (two with persistence of radiological abnormalities and one with a relapse of symptoms).

## 5. Discussion

*Bartonella henselae* is a Gram-negative organism that should be included in the differential diagnosis of localized lymphadenitis in an immunocompetent host. It is usually acquired through a scratch, bite, or intimate contact with cats, especially kittens, although a case after dog scratch has been described [38, 39].

In 90% of cases, CSD manifestation is represented by subacute, localized, self-limited lymphadenitis preceded by local cutaneous reaction at the scratch site. Resolution of symptoms usually occurs within 2–4 weeks [3]. Lymphadenopathy is usually self-limited, not requiring antibiotic therapy.

About 10% of patients with CSD show atypical manifestations including prolonged course of fever (i.e., more than two weeks), erythema nodosum, and hepatic and splenic granulomas [3, 4, 40]. In the literature, bone involvement during CSD is a rare manifestation accounting for 0.17–0.27% of all CSD cases [3, 4].

No specific factors influencing the spreading from localized to disseminated infection have been recognized so far [41].

In this report, 52 CSDs with osteomyelitis in immunocompetent children have been analyzed.

While osteomyelitis caused by other common organisms, such as *Staphylococcus aureus*, usually occurs in males younger than five years of age [42, 43], the average age of patients with *Bartonella* osteomyelitis was 7.8 years with an equal sex distribution, confirming previous reports by Hajjaji et al. [41].

The study by Maman et al. investigated CSD musculoskeletal manifestations in children and adults. This study reported only two cases of CSD osteomyelitis, both in children, indicating that children may be at higher risk of bone complications compared to the general population [41, 44].

Our case review indicates that fever, often with a prolonged course, osteoarticular pain, and functional impairment are the most common symptoms in *Bartonella* osteomyelitis.

Superficial lymphadenopathy, usually considered the hallmark of CSD, is less frequent, making early etiological diagnosis rather difficult. While swelling of tissues around the bone affected area is frequently present, signs such as rubor and calor are extremely rare.

In agreement with Hajjaji et al. in 2007 [41], we found that primary osteomyelitis with no other systemic manifestation was the most common presentation.

Nonspecific findings of CSD including abdominal pain, fatigue, night sweats, and weight loss were detected in 34.7% of children. In these cases, differential diagnosis is required to distinguish disseminated CSD from diseases with worse prognosis such as tuberculosis, chronic granulomatosis disease, histiocytosis, lymphoma, and malignancy [45].

Furthermore, while osteomyelitis caused by other common organisms mainly involves the legs [46], we found that the most affected bones during CSD are vertebral bodies followed by limbs [41, 45, 47]. Osteomyelitis of the skull is a rare manifestation with 10 cases in 50 years in the literature [48–50].

Most patients presented with a solitary bone lesion; no strict correlation has been found with multiorgan involvement and multifocal osteomyelitis, since only 37.5% of patients with disseminated CSD had a multifocal osteomyelitis.

CSD infection disseminates in three ways: hematogenous, lymphatic, or contiguous [51]. Observing lymphadenopathy distribution in relation to the osteomyelitis site, in contrast with what was reported by Robson et al. [52], only 39.3% of lymph nodes were contiguous or in a drainage area of the bone infected site in this review, indicating potentially more hematogenous dissemination of CSD in children.

Skin test and histology on biopsied lesion were the preferred diagnostic tools before the advent of serology.

Skin test has high sensitivity but poor standardization and is not approved by the US Food and Drug Administration because its preparation derives from lymph nodes pus of patients with CSD [53].

Since *Bartonella* species may require from 1 to 4 weeks of the incubation period and bacterial isolation is usually unsuccessful [51, 54], culture does not represent the best microbiologic test for diagnosis.

After its introduction, serology became the best diagnostic tool for CSD, given its high sensitivity (88%) and specificity (97%) [41].

The most frequently used serologic methods are indirect fluorescence assay (IFA) and enzyme immunoassay (EIA) [51]. Positive IgM titer strongly suggests acute disease, but IgM production is usually brief. IgG titers usually indicate current or recent *Bartonella* infection, even if sensitivity appears suboptimal and the prevalence of positive *Bartonella* serology in the general population is 4–6% creating false positive tests [55, 56].

In our laboratory, we have an immunofluorescence assay without possibility to obtain IgM and IgG titers; for this reason, we were not able to document a fourfold increase in antibody titers, but we observed IgM becoming negative while IgG persisting positive after one month from previous serology.

PCR involves amplification of *Bartonella* species genes (16S rRNA gene, citrate synthase gene (gltA), and htrA gene) directly from tissue or aspirate. It is specific but with a variable sensitivity (40–70%) with high risk of negative results if sample collection occurs too early (<6 weeks) [47, 57–61]. PCR testing of the lymph node was not performed in our case since it is an invasive procedure and requires sedation in pediatric patients.

To detect bone involvement in our review, there was a slight preference for MRI and bone scintigraphy, and usually, a combination of two or more radiological exams had to be applied for diagnosis.

Scintigraphy is a useful method to evaluate the presence of multifocal disease, while MRI, for its excellent sensitivity and specificity, is currently the best technique to find early lesions (especially in vertebral bodies) and soft tissue damage [62–65]. Therefore, the combination of MRI and scintigraphy could represent the best way to detect possible multiple lesions and to thoroughly study the involved bone and surrounding tissue.

Treatment of CSD osteomyelitis involves mainly medical intervention [46], although the use of antibiotics for CSD infection in immunocompetent children is still controversial. The only one prospective double-blind, placebo-controlled trial designed for treatment of *Bartonella* infections included only uncomplicated CSD. In this study, Bass et al. reported a decrease of 80% of the initial lymph node volume in 7 of 14 azithromycin-treated patients and only 1 of 15 placebo-treated controls during the first 30 days of observation (*P*=0.026) [1].

Various antibiotic regimens, most including agents with in vitro activity against *Bartonella* species, have been prescribed in patients with CSD bone involvement: macrolides, rifampicin, beta-lactams, TMP-SMX, aminoglycosides, quinolones, clindamycin, and tetracycline. Only gentamicin and rifampin appear bactericidal [66, 67], and a retrospective review of 202 patients indicated rifampin, ciprofloxacin, gentamicin, and TMP-SMX as the four most efficacious antibiotics [68]. In our review, it was unclear whether the combination of rifampicin and TMP-SMX was more effective than azithromycin alone or the lesion would have healed anyway without further therapy. 11 patients who received neither medical therapy nor surgery had good prognosis.

Due to its rareness, no randomized, prospective controlled trial has been performed to establish the preferred antibiotic regimen for CSD osteomyelitis [69]. Indeed, the role of antibiotics in the improvement in disseminated disease mainly derives from retrospective reviews [70, 71].

In most of those cases, antibiotic therapy was combined or sequential. Differently from what has been previously reported, antibiotic monotherapy has been chosen in only 38.5% of cases.

Despite recommended duration of therapy for osteomyelitis of 4–6 weeks [46], average duration in this review for CSD osteomyelitis was 3 weeks, which could reflect the self-limited nature of the disease and atypical manifestations like bone involvement.

Fifty-eight percent of children received a surgical procedure. In more than a half, a diagnostic biopsy had been performed, with a high prevalence of bone biopsy. After diagnostic biopsy, most patients were treated with medical therapy.

As previously reported by Mirouse et al., surgical management was rare, mostly occurring after complications such as an epidural abscess or severe skeletal or articular involvement [72].

According to the literature, most of patients had an excellent prognosis [1, 41, 52, 71, 73, 74] with a radiological recovery with an average time of 4 months.

## 6. Conclusions

Osteoarticular pain or limitation during CSD in children should always be investigated for the possibility of *Bartonella* bone spreading even when there are no other signs or symptoms of systemic dissemination.

Currently, serology represents the gold standard for diagnosis. Valuable support could be offered by PCR testing of peripheral lymph nodes, in case of concomitant lymphadenopathy. Owing to good prognosis of *Bartonella* osteomyelitis, invasive procedures to obtain the bone material, as proposed in the past, should be avoided.

MRI is the best radiographic technique to define early lesions and spreading of infection in the surrounding tissues without radiation exposure.

Despite its wide use, antibiotic therapy in CSD osteomyelitis remains a never-ending dilemma, and more studies are needed especially to define, if really needed, the best antimicrobial regimen and treatment duration.

## Figures and Tables

**Table 1:** Pediatric cases of osteomyelitis related to *Bartonella henselae* infection.

Cases of osteomyelitis	*N*=51
*Epidemiology*	
Age (years)	7.8 (±3.8)
M : F	1 : 1
Contact with cats	91.8%
*Clinical presentation*	
Fever	84.3%
Fever (lasting >2 weeks)	64.7%
Lymphadenopathy	64.7%
Cervical	45.5%
Axillary	15.6%
Inguinal	12.1%
Osteoarticular pain	88.2%
Functional impairment	40%
Swelling	37.3%
Systemic signs^∗^	34.7%
Site of osteomyelitis	
Vertebral bodies	51%
Limbs	32.7%
Skull	19%
Unifocal	73.1%
Multifocal	26.9%
*Imaging*	
X-ray	50%
MRI	50%
CT	46.2%
Scintigraphy	48.1%
*Diagnosis*	
Serology	78.4%
PCR	27.5%
Biopsy	29.4%
*Treatment*	
Medical	66%
Macrolides	46.2%
Rifampicin	33.3%
Beta-lactams	30.8%
TMP-SMX	23.1%
Aminoglycosides	23.1%
Median duration	23 days
Surgery	20.8%
None	12.5%
*Outcome*	
Rapid resolution (<2 weeks)	43%
Prolonged symptoms	57.5%
Incomplete recovery	7.7%

^∗^Abdominal pain, fatigue, night sweat, and weight loss.

## Data Availability

The datasets used and/or analyzed during the current study are available from the corresponding author on reasonable request.

## Abbreviations

CSD: Cat-scratch disease
US: Ultrasound
MRI: Magnetic resonance imaging
TMP-SMX: Trimethoprim-sulfamethoxazole
CT: Computed tomography
XR: X-ray
PCR: Polymerase chain reaction
IFA: Indirect fluorescence assay
EIA: Enzyme immunoassay.

## Supplementary Materials

Supplementary Materials Pediatric cases of osteomyelitis related to *Bartonella henselae* infection.
