# Supplementary material for: Osteomyelitis in Cat-Scratch Disease: A Never-Ending Dilemma—A Case Report and Literature Review
Source: Case Rep Pediatr. 2018 Apr 1;2018:1679306. doi: 10.1155/2018/1679306 (PMC5902065; doi:10.1155/2018/1679306)
Supplement: Supplementary Materials — Pediatric cases of osteomyelitis related to Bartonella henselae infection. [file 1679306.f1.docx]

**Supplementary material: Pediatric cases of osteomyelitis related to *Bartonella henselae* infection**
